# Laparoscopic repair of the caesarean section scar niche: A prospective cohort study

**DOI:** 10.1371/journal.pone.0318592

**Published:** 2025-07-02

**Authors:** Anna Abacjew-Chmylko, Dariusz G. Wydra, Hanna Olszewska, Sambor Sawicki, Katarzyna Stefanska

**Affiliations:** Department of Gynecology and Obstetrics, Medical University of Gdansk, Gdansk, Poland; Kasr Alainy Medical School, Cairo University, EGYPT

## Abstract

**Objective:**

To evaluate the effect of laparoscopic repair of the large niche on short-term and long-term outcomes, i.e., extent of scar healing (increase in scar thickness or residual myometrium (RM) and decrease in niche depth), decrease in menstrual symptoms, likelihood of conception and successful delivery.

**Study design:**

A prospective observational cohort study.

**Methods and findings:**

Among 333 patients referred with a niche diagnosed in transvaginal ultrasound (TVU), a group of 127 met the selection criteria for repair surgery (RM of <2.5 mm in its thinnest part in hysterosonography (HySoG) and a desire to conceive) and underwent the laparoscopic procedure (uterine cesarean scar and niche walls cold knife resection followed by resuturing of the uterine wall) between November 2015 and October 2022.

The laparoscopic repair of niche increased the RM to 6.5 ± 2.6 mm in TVU and 6.1 ± 2.5 mm in HySoG. Postoperative failure, defined as incomplete scar formation with a niche and residual myometrium thickness under 2.5 mm, occurred in 8.2% of cases. Furthermore, 20.9% of scars showed residual myometrium thickness below 4 mm. Conversely, the rate of postoperative diverticulum, defined as an indentation at the site of the cesarean section scar with a depth of at least 2 mm, was 70.9%. Furthermore, indentations greater than 3 mm were found in 49.1% of cases.

The surgical procedure significantly alleviated symptoms related to the niche: duration of postmenstrual spotting (*P* < 0.001), length of menstrual bleeding (*P* = 0.03), menstrual pain (*P* < 0.001) and menstrual flow (*P* = 0.02).

In patients with a sustained postoperatively desire to conceive (N = 79, 62.2%) a vast majority fulfilled childbearing plans (n = 42, 53.2%), for at least once (93%). The best surgical outcomes were obtained when the procedure was performed in the follicular phase of the menstrual cycle before the peri-ovulatory time (*P* = 0.02) and the uterine reconstruction was employed with double-layer horizontal mattress sutures.

**Conclusions:**

The conducted study demonstrated that the surgical procedure for scar repair brings benefits by reducing clinical symptoms of the defect, improving scar parameters, and achieving a high rate of successful reproductive plans.

## Introduction

Niche, also known as a uterine diverticulum, isthmocele, or caesarean scar defect (CSD), basing on the international consensus presented by Jordans et al. [1], is defined, as an indentation at the site of the CS scar with a depth of at least 2 mm. Previous research described the niche as a myometrial defect or discontinuity of the caesarean section scar that either adjacent to the cervical canal [2,3] or from the external uterine wall (from the serosal surface) or a combination of the above [4].

The causes of this insufficiency in caesarean scar healing are multifactorial and are not yet fully understood [5].

The objective of the laparoscopic repair of the caesarean section scar niche is to reconstruct the myometrial defect and create sufficient myometrial thickness. While there are currently no randomized controlled trials to definitively validate the benefits of the repair in question, it holds the potential to alleviate various obstetrical complications. These may include:

(a) infertility or problems with embryo implantation due to fluid accumulation [6,7],

(b) gestational sac implantation in the diverticulum,

(c) complications associated with placenta accreta spectrum (PAS), and

(d) higher risk of uterine dehiscence during pregnancy or uterine contractions [8–14].

However, the probability of these consequences is only assumptive as there is little data comparing obstetrical outcomes based on the repair status [14].

Treatments for caesarean scar defects include laparoscopy, laparotomy, and transvaginal procedures [15]. Since the Nezhat group performed the first laparoscopic niche repair in 2003 [16], most studies have been limited to small groups of patients, with only a few reporting the effects of laparoscopic caesarean scar defects [16–21], and the largest series reporting on 101 patients by Vervoort et al. [22]. The eligibility criteria for laparoscopic niche repair in these studies included: (a) a desire to conceive [18–22], (b) the presence of large niches with a residual myometrium (RM) of less than 3 mm [18–22], (c) the presence of CSD symptoms as: abnormal uterine bleeding (AUB) [18–22], chronic pelvic pain or dysmenorrhea [18–20,22], secondary infertility [18–20,22] or midcycle intrauterine fluid accumulation [22].

This prospective study aims to evaluate the effect of laparoscopic repair of the niche on short-term and long-term outcomes, including the degree of scar healing, reduction in menstrual symptoms, likelihood of conception, and successful delivery.

## Methods

A prospective cohort study was conducted on 333 patients with caesarean section scar niche referred to the Department of Gynecology and Obstetrics at the Medical University of Gdansk, Poland, between August 20^th^, 2015, and June 28^th^, 2022. Based on hysterosonography performed in our clinic, 162 patients met the selection criteria and were qualified for laparoscopic repair. Finally, 127 patients underwent treatment between November 2^nd^ 2015 and October 31^st^ 2022. The patients provided their informed written consent for treatment. A flow chart detailing the selection of patients is shown in the S1 Fig.

The selection criteria for surgery included:

1. the presence of a niche with residual myometrium (RM) of less than 2.5 mm in its thinnest part in HySoG,
2. a desire to conceive and
3. having a niche-associated symptom.

The niche was considered when an ultrasonographic hypoechogenic area in the caesarean scar region was observed, and a patient experienced symptoms such as postmenstrual uterine bleeding, abdominal pain, infertility, or only midcycle fluid accumulation in the scar.

Patients with RM of less than 2.5 mm and postmenstrual spotting but without a desire to conceive were recommended for hormonal treatment in the form of levonorgestrel IUD or oral contraception and/or hysteroscopic resection of the edges of the niche without coagulating its base due to increased risk of perforation.

The exclusion criteria included age < 18 years and >45 years (n = 2), pregnancy (n = 3), medical contraindications for laparoscopy or general anaesthesia (n = 2), coexisting conditions requiring emergency treatment (n = 1), suspicion of cervical neoplasia, uterine, or ovarian cancer (n = 2), abnormal, heavy uterine bleeding requiring diagnostic examination (n = 2), and lack of informed consent for clinical treatment (n = 6).

Patients who eventually declined the intention to conceive once they received complete information about the treatment procedure and postoperative recommendations and after a complete diagnostic evaluation had their consent to treatment withheld. They were informed of the possible increased risk of niche-related complications in subsequent pregnancies.

**Fig 1:**
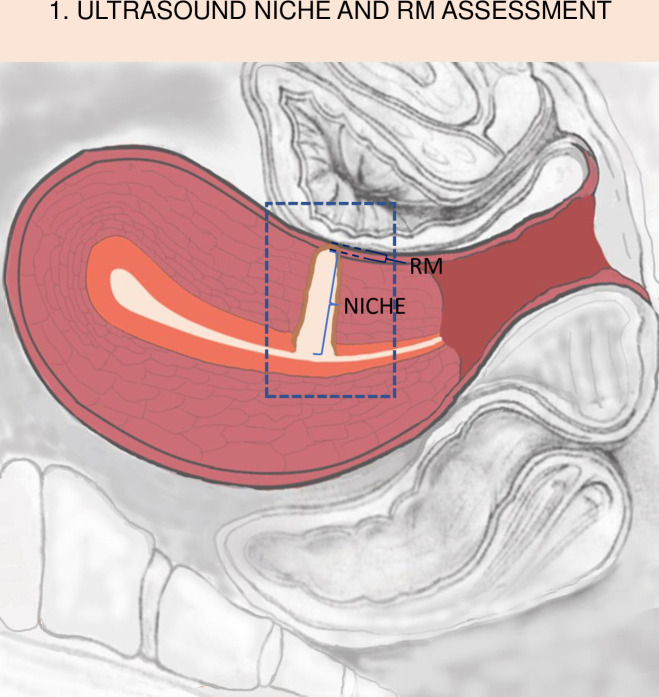
The process of ultrasonographic niche assessment – measurement of the cesarean section scar NICHE depth and the residual myometrial thickness (RM).

Transvaginal ultrasound (TVU) was the preliminary diagnostic tool for identifying the isthmocele, while hysterosonography (HySoG) was the definitive one [2,3]. In both examinations, the thickness of the uterine scar at the thinnest point in the sagittal plane (RM, residual myometrium) and the corresponding depth of the isthmocele (NICHE) were measured (Fig 1). This assessment was conducted both before the surgery (pre-RM TVU, pre-NICHE TVU, pre-RM HySoG, pre-NICHE HySoG) and after the surgery (post-RM TVU, post-NICHE TVU, post-RM HySoG, post-NICHE HySoG), all in accordance with established standards [1–4]. In the postoperative assessment, the evaluation of the scar in hysterosonography was omitted when the amount of fluid with or without mucus retained in the cervical canal and within the scar was deemed sufficient for TVU scar assessment. Both preoperative and postoperative examinations were performed by two ultrasonographers (AAC, HO). The ultrasonographers were blinded for the type of surgical technique used.

The procedure for repairing the uterine scar was performed laparoscopically by three experienced laparoscopic gynaecologists (AAC, DGW, SS). This surgical technique was developed in 2016 based on limited literature data [17,23–25] and the Department’s experience for suturing the uterine wall after a myomectomy and caesarean section. Although similar to techniques used in prior studies [19,20,22,26], this method differed in the intraoperative identification of the defect, excision of the scar along with the defect, and uterine suturing.

In the largest initial cohort study by Donnez et al. [17], which included 38 patients, the niche was identified using a Hegar probe, then the niche was removed using a CO_2_ laser, and three layers of sutures were applied (two single layers and a third continuous layer of sutures). In a similar quantitative study by Karampelas et al. [20], the defect was identified on a probe, the scar was excised using a CO_2_ laser or cold scissors, and the uterus was sutured in two layers – with three separate X-sutures in the deeper part of the defect and a superficial layer of a running suture. The largest study to date, with 101 patients, was described by Vervoort et al. [22] based on the technique presented by Huirne et al. [26], in which the cold knife resection technique was similar to ours, except that the niche was always identified with a hysteroscope, the bladder was dissected only after filling it with methylene blue, single sutures covering the entire scar thickness were used, and an additional outer layer of sutures was applied to reduce tension within the scar. Huirne et al. [26], like Donnez et al. [17] and Karampelas et al. [20], used the technique of suspension of the round ligaments (Baldy anterior) in cases of extremely retroverted uterus. Additionally, Huirne et al. [26] applied an adhesion barrier agent.

**Fig 2:**
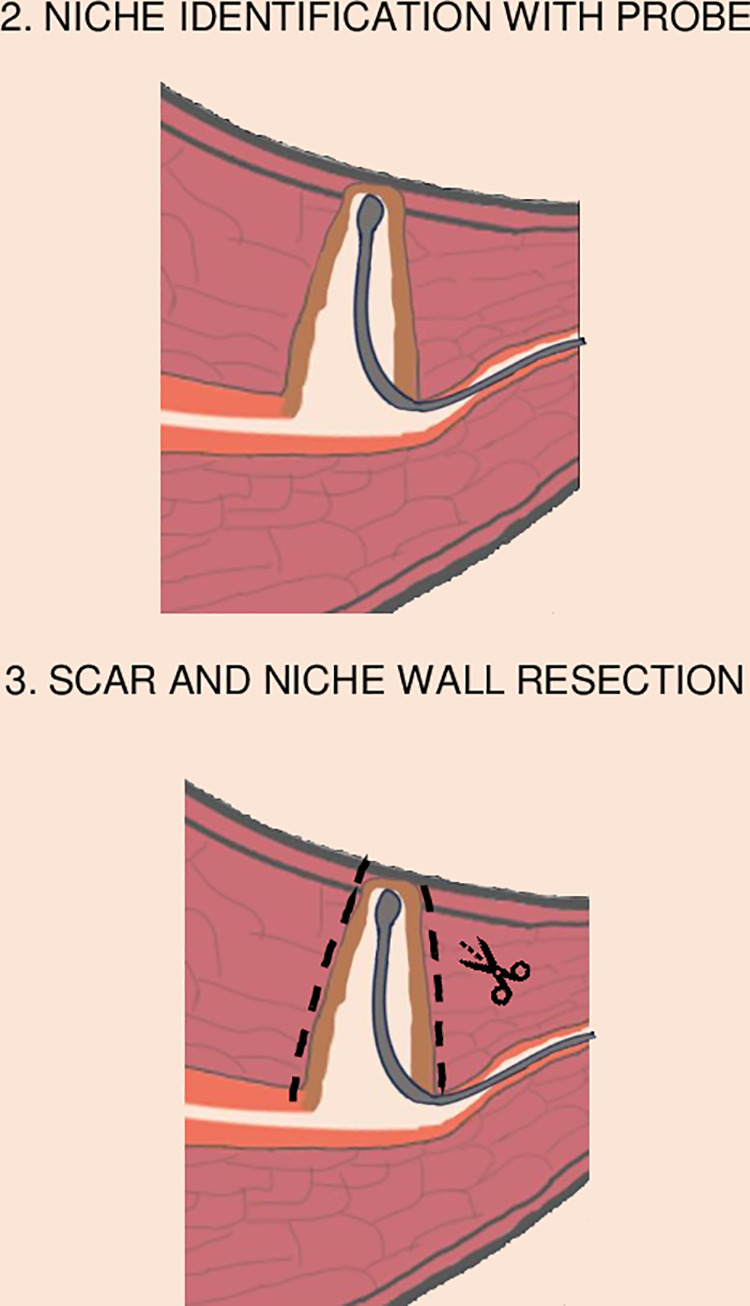
The initial steps of surgical caesarean scar repair.

**Fig 3:**
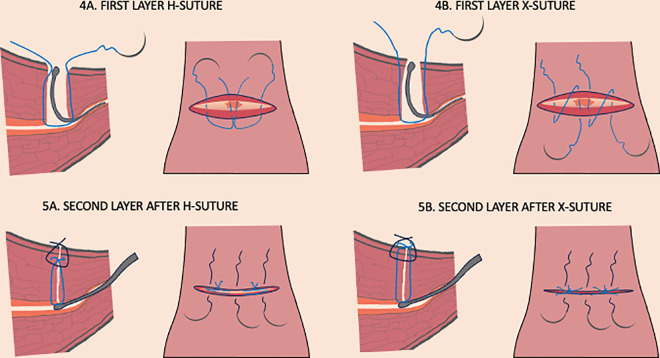
The procedure of uterine suturing during the surgical caesarean scar repair.

The technique used this research involved identifying the niche with a uterine probe or, a hysteroscope if there was a problem with identification, incising the uterus above the thinnest point of the defect, excising the remaining scar (Fig 2), and resuturing the uterine wall, as detailed in S1 Table. The suturing modes used were single-layer cross-mattress sutures (X-suture, single layer), double-layer cross-mattress sutures (X-suture, double layers), or double-layer horizontal mattress sutures (H-sutures, double layers) (Fig 3). The double-layer technique with the H-sutures technique employs a suturing method in which the first layer is positioned at approximately three-quarters of the scar’s thickness. This initial layer serves a dual purpose: it enhances the alignment of the endometrium while also providing support for the deeper myometrial layers. By anchoring the endometrium and deep layers more effectively, it promotes optimal healing and integration of the tissue. In addition to this, the external sutures play a crucial role by bringing the outer layer of the uterus together. Beyond merely closing the incision, these sutures are thought to help alleviate tension within the uterus. This reduction in tension is significant, as it may prevent the uterus from developing excessive retroflexion, a condition that can lead to niche formation [1–3]. Overall, the double-layer technique is designed to enhance both the structural integrity and functional recovery of the uterus following surgery.

1. Identification of the niche during the laparoscopic procedure with a uterine probe that is positioned in the base of the niche.
2. Cold knife resection of the cesarean scar with the niche walls, surrounding cicatricial tissue and residual myometrium.
3. In the first layer two H-sutures are placed at 3/4 thickness of the uterine wall. 5A. In the second layer three single sutures are placed on the external part of the wall overlapping the first layer H-sutures sutures.
4. In the first layer two X-suture are placed at full thickness of the uterine wall. 5B. In the second layer three single sutures are placed on the external part of the wall overlapping the first layer X-sutures.

Early treatment outcomes were assessed between 3–6 months post-surgery and included evaluating the depth of the defect (post-NICHE) and the thickness of the RM (post-RM), changes in menstruation features as the duration of postmenstrual spotting, total duration of bleeding (the sum of menstrual flow and spotting), pain intensity (on a two-level scale), and severity of menstrual bleeding (on a three-level scale). Postmenstrual spotting was defined as either ≥2 days of red spotting or ≥1 day of brownish discharge directly after the menstrual period or a few days later. Two parameters were also calculated: what fraction of the uterine bleeding was taken by spotting (spotting length compared to uterine bleeding length) and what part of the cycle was taken by uterine bleeding (uterine bleeding length in the cycle).

Long-term outcomes, fulfilling pregnancy plans, were evaluated by phone or in person at scheduled intervals (every 6 months) – until the completion of reproductive plans or until the end of the follow-up period, which was on December 12^th^, 2022, or an average of 32.4 months (±13.3).

Patients were informed of the need to wait at least 6 months before attempting to become pregnant and that delivery should be by caesarean section.

All patients diagnosed with endometriosis during surgery had the affected areas resected and were given contraceptives for the next six months, if no contradictions were found, provided there were no contraindications.

The study used the student-T test to compare groups for statistical differences. For the univariate assessment of the relationship between dependent and independent variables, the following tests were employed: ANOVA, linear regression analysis, and logistic regression analysis, depending on the nature of the data.

The study was approved by the Bioethics Committee for Scientific Research, Medical University of Gdansk, Poland (approval number NKBBN/297/2015) on the 17.08.2015 and it covered the prevalence of the niche, the ultrasonographic assessment and the surgical repair procedure.

## Results

The clinical characteristics of 127 patients and the circumstances of the surgical procedure who underwent laparoscopic repair of the niche are presented in Tables 1 and 2. The patients had an average age was 33.2 years (±4.4), with 20% being below 30, 46.2% aged between 30 to 34.9 years, and 26.0% aged between 35 to 39.9 years. Only 5.5% of the patients were aged 40 years or more. Among the patients with the caesarean section scar niche, 72.4% had undergone a C-section once in their lives, 26.8% had previously undergone two C-sections, while 0.8% had undergone previously three caesarean deliveries. The diagnosis of CSD was made within two years of the last caesarean section in 22% of the patients, while in 32% of the patients, the diagnosis was made at least five years after the last procedure. The infertility was reported by 34 patients, with a mean duration of 3 years (ranging from 1 to 5 years). Five patients had previously undergone assisted reproductive treatment procedures.

**Table 1:** Basic characteristics of patients undergoing surgical treatment for a cesarean section scar niche.

*Feature of patients*	*n = 127*
**Age (years)**	33.2 ± 4.4 (18.9-43.7)
**Time since the last caesarean section (years)**	4.4 ± 3.1 (0.4-17.3)
**Birth history**	
Number of all deliveries	1.3 ± 0.5 (1-3)
Vaginal delivery following a C-section	4 (3.1%)
C-section following a vaginal delivery	4 (3.1%)
Miscarriage following a C-section	18 (14.2%)
**BMI (kg/m**^**2**^)	24.1 ± 3.9 (18-36.3)
Overweight*	33 (26.0%)
Obesity*	12 (9.4%)
**Symptoms reported by patients ^**	
Postmenstrual spotting	99 (78.0%)
Abdominal pain during menstruation	41 (32.3%)
Infertility	34 (26.8%)
No symptoms – only intrauterine fluid accumulation	6 (4.7%)
**Perimenstrual symptoms**	
Postmenstrual spotting	102 (80.3%)
Postmenstrual spotting ≥2 days	87 (68.5%)
Dysmenorrhea	72 (57.6%)
**Hormonal contraception**	18 (14.2%)
**Chronic diseases**	
Immune-related disorders or haematological disorders	8 (6.5%)
Hypothyroidism during treatment	35 (19.9%)

Data are reported as: mean ±SD (range) or n (%).

*classification of overweight and obesity by WHO.

**^** Several symptoms could have been present in a single patient.

**Table 2:** The characteristics of the performed surgical procedure.

Features of surgical procedure	n = 127
**Technique**	
H-sutures, double layers	33 (26.0%)
X-sutures, double layers	75 (59.1%)
X-sutures, single layer	19 (15.0%)
**Suture layers**	
Double	94 (85.0%)
Single	19 (15.0%)
**Type of deep layer sutures**	
H-sutures	33 (26.0%)
X-sutures	94 (74.0%)
**Pelvic adhesions**	52 (40.9%)
**Adhesion of the uterus to the anterior abdominal wall**	30 (23.6%)
**Endometriosis**	15 (11.8%)
**Phase of the menstrual cycle during the procedure**	
Follicular phase	47 (39.5%)
Ovulation period	36 (30.3%)
Luteal phase	36 (30.3%)

Data are reported as: n (%).

Significant changes in menstrual patterns and uterine bleeding were observed in the postoperative assessment of patients, as shown in Table 3. In a retrospective analysis of individual symptoms, the criteria of the Caesarean scar disorder reported by Klein Mauleman et al. [27] were fulfilled by 97.5% of patients. All of the patients experienced at least one of the primary symptoms: postmenstrual spotting, pain during uterine bleeding, or unexplained secondary infertility alongside the presence of intrauterine fluid. However, this analysis was limited because we were unable to capture all of the reported symptoms of CSD, particularly the secondary ones, from the patients’ reports.

**Table 3:** Change in the character of menstruation before and after the surgery.

Feature of menstruation	Before	After	*P*-value
Menstrual bleeding length (days)	5.0 ± 1.7 (2-14)	4.7 ± 1.3 (2-7)	**0.03**
Postmenstrual spotting length (days)	6.0 ± 6.3 (0-27)	1.7 ± 3.3 (0-25)	**<0.001**
Uterine bleeding length (days)	11.0 ± 6.3 (3-30)	6.3 ± 3.3 (2-30)	**<0.001**
Spotting length compared to uterine bleeding length (%)	42.9 ± 27.6 (0-90)	18.0 ± 22.9 (0-83.3)	**<0.001**
Uterine bleeding length in cycle (%)	37.7 ± 21.5 (13-100)	22.1 ± 11.3 (7-100)	**<0.001**
Dysmenorrhea (%)	72 (57.6)	30 (30.0)	**<0.001**
Heavy menstrual bleeding (%)	56 (44.4)	32 (34.3)	**0.02**

Data are reported as: mean±SD (min – max) or n (%)

### Pregnancy achievement

The primary eligibility criterion for surgical treatment was a desire to conceive. However, 26.0% of patients abandoned their reproductive plans directly after the procedure, and contact was lost with 4 patients. In addition, 11 patients did not achieve a sufficiently long postoperative observation period (exceeding 6 months) to begin attempting pregnancy. Thus, a complete follow-up regarding pregnancy outcomes was conducted for 79 patients (62.2%), and 42 participants achieved a total of 46 pregnancies resulting in childbirth (53.2%). Among the 46 pregnancies, 43 were delivered by caesarean section, while three were delivered vaginally. Of all the patients, 39 had single births, 2 had two births each, and 1 had three births, with all but three deliveries by C-section. There were no cases of uterine rupture or dehiscence of the scar, nor were there any patients with placenta accreta spectrum. All cesarean sections were uneventful except for one during which a uterine hemorrhage occurred, necessitating an emergency hysterectomy. Upon pathological examination, an aneurysmal dilation of vessels was discovered in association with placenta previa. The patient had a history of two C-sections before the niche laparoscopic repair and a uterine rupture during the second delivery. Two women delivered prematurely at 36 weeks of gestation due to cervical insufficiency and pPROM. Furthermore, 25 women (19.7%) abandoned their reproductive plans after an interval of unsuccessful attempts, ranging from 10.8 to 56.9 months (median 34, ICR 28.9–44.8), while 12 (9.4%) were still actively trying to conceive at the end of follow-up.

Miscarriages (not including failed IVF procedures) were noted in 11 patients (13.9%), while there were no cases of a niche ectopic pregnancy.

Postoperative changes in the uterine C-section scar structure were significant, with an increase in uterine scar thickness and a decrease in the depth of the niche, as shown in Table 4. Both the basic TVU assessment and the use of HySoG indicated these changes. Incomplete scar formation, defined as a post-caesarean RM measurement of <2.5 mm, was observed in 8.2% (n = 9) of the patients postoperatively, while the prevalence of scars with post-RM < 4 mm, was 20.9% (n = 23). The scar diverticulum “reoccurred” in 70.9% of women (n = 78) when using the criterion of post-NICHE >2 mm, whereas with the criterion of post-NICHE >3 mm, it occurred in 49.1% of cases (n = 54). The learning curve associated with the procedure has proven to be beneficial in decreasing the failure rate over time. In the first four years, while performing 64 procedures, the failure rate was 10.9%, with seven cases showing a postoperative niche with a RM of less than 2.5 mm. In the subsequent four years, during which we conducted 63 cases, only two such incomplete scar formations were recorded, resulting in a reduced rate of 3.2%.

**Table 4:** Comparison of RM thickness and NICHE depth pre- and post-operatively measured in transvaginal ultrasonography (TVU) and hysterosonography (HySoG).

Niche features	Preoperatively N = 127 (pre- TVU and pre- HySoG)	Postoperatively N = 110 (post- TVU) N = 77 (post- HySoG)	*P*-value	95% Confidence Intervals	
				Lower	Upper
RM (TVU)	2.3 ± 1.6 (0-8); 1.9 (1.3-3.0)	6.5 ± 2.6 (1-14); 6.6 (4.8-8.2)	**<0.001**	3.68	4.72
RM (HySoG)	1.2 ± 0.5 (0-2.4); 1.2 (0.96-1.5)	6.1 ± 2.5 (0-12); 6.3 (4.5-7.8)	**<0.001**	4.28	5.43
NICHE (TVU)	7.5 ± 4.0 (0-20); 7.3 (4.8-10.0)	3.1 ± 2.4 (0-10); 2.7 (1.4-4.7)	**<0.001**	−5.28	−3.48
NICHE (HySoG)	9.6 ± 3.7 (0-20); 9.5 (7.4-12.0)	4.3 ± 2.7 (0-12); 4.0 (2.2-6.3)	**<0.001**	−6.07	−4.09

Mean ±SD (min – max); med. (ICR1-ICR3).

### Additional procedures

Thirteen patients underwent additional procedures due to recurrent abnormal uterine bleeding observed over time. Among these patients, nine were eligible for hysteroscopic resection because they experienced a small postoperative niche. Additionally, four patients required a repeat laparoscopic scar repair due to a large niche with a RM of less than 2.5 mm.

### Surgical technique

The type of surgical technique employed for uterine scar repair after caesarean section has been shown to have a significant impact on the postoperative recurrence of a diverticulum. Specifically, the type of suture utilized – whether double layer horizontal mattress sutures (H, double), double layer cross-mattress sutures (X, double), or single layer cross-mattress sutures (X, single) – was found to affect the frequency of post-NICHE defined as >3 mm (OR 0.22 95% CI, 0.16–0.66, *P* < 0.001) or defined as >2 mm (OR 0.45 95% CI, 0.24–0.89 *P* = 0.02) and the depth of the post-NICHE (*P* = 0.003), both assessed exclusively in TVU (*P* = 0.01) and HySoG (*P* = 0.02) (S2 Table).

The postoperative recurrence rate of the niche formation, defined as an indentation greater than 3 mm, was the lowest with the H-double technique (21.4%), in contrast with other techniques where the recurrence rate was above 50%. The depth of the niche in the scar was the smallest when the H-suture double layer techniques were applied, with no statistical differences if 2 or 3 H-sutures were used (*P* > 0.05, Bonferroni analysis). However, neither the type of uterine suturing technique during the procedure nor the number of suture layers had any impact on the absolute postoperative RM (scar thickness) values (S2 Table).

Notably, neither of the surgical aspects of the repair technique had an impact on the characteristics of uterine bleeding, including the length of postmenstrual spotting, length of menstruation, menstrual pain, or menstrual flow.

Regression models were considered to assess the impact of procedure type on pregnancy outcomes; however, no statistically significant associations were observed between the type of procedure and the likelihood of conception or successful childbirth.

### Perioperative features

The timing of the surgical procedure in relation to the menstrual cycle was found to have an effect on postoperative RM values. The results indicated that the further the procedure was carried out in the menstrual cycle, the smaller the increase in the achieved RM gain (∆ post-RM – pre-RM HySoG, *P* = 0.02, estimate = −0.07 for cycle days), and thus the highest RM values were obtained in the follicular phase (*P* = 0.02). In contrast, procedure failure (RM < 2.5) only occurred when the procedure was performed during the periovulatory or luteal phase of the cycle (*P* = 0.049). However, no observed influence of the cycle phase on postoperative NICHE depth was found.

During the surgical procedure, adhesions between the uterus and the anterior abdominal wall were released in every case to gain access to the location of the uterine scar from the caesarean section, but only to the extent necessary for uterine scar repair. Results showed that the presence of abdominal adhesions correlated with a higher frequency of postoperative niche formation (81.8% vs 63.6%, *P* = 0.04, OR 2.6; 95% CI, 1.02–6.43), worse postoperative scar thickness results (post-RM HySoG thickness, 5.34 ± 2.38 mm vs. 6.71 ± 2.52 mm, *P* = 0.02), and a statistically more minor increase in postoperative scar thickness (∆ post-RM – pre-RM HySoG, 4.1 ± 2.37 mm vs. 5.43 ± 2.51 mm, *P* = 0.049). However, the adhesions did not influence the analyzed parameters.

The intraoperative diagnosis of endometriosis did not influence the results of the analyzed parameters.

None of the analyzed parameters, including the surgical technique day or phase of the menstrual cycle at the time of the procedure, the presence of adhesions, or endometriosis, ultimately influenced the outcome of conceiving or childbirth (*P* > 0.05).

## Discussion

This study stands as the first comprehensive analysis comparing surgical techniques and perioperative factors in laparoscopic repair of uterine scar defects, providing valuable insights into early and late pregnancy-related outcomes.

### Strengths and limitations

A notable limitation of our paper is the lack of randomization in the selection of treatment methods and the different proportions of groups, which introduces large bias in comparing surgical techniques. However, the choice of technique was driven more by the surgeon’s discretion than by anatomical conditions, suggesting that our findings carry a stronger weight of scientific evidence. Additionally, the groups analyzed were rather sizable, therefore driving preliminary conclusions on the surgical methods is feasible and becomes an implication for the need of performing a study with randomization.

Moreover, this research contributes to one of the few long-term assessments concerning postoperative pregnancy outcomes, as most prior studies lack extended observation periods. For instance, childbirth rates were notably higher in our study compared to findings by Karampelas et al. (35.5%) [20] and Donnez et al. (21.1%) [22]. Nonetheless, further in-depth studies are necessary to evaluate the relationship between surgical techniques, pregnancy outcomes, improvements in scar parameters, and menstrual bleeding patterns.

An important limitation in analyzing postoperative conception rates in our cohort is the limited access to—and acceptance of—assisted reproductive technology (ART) in Poland. During the period when most procedures were performed, ART was neither reimbursed by a national public program nor covered by private insurance. Consequently, patients bore the full financial burden of treatment, which frequently led to discontinuation or avoidance of ART altogether. Additionally, a substantial proportion of patients declined in vitro fertilization for religious reasons. Together, these factors complicate our assessment of infertility rates by condition and skew the observed postoperative conception outcomes.

### Interpretation

To minimize the potential for overtreatment in our patients with uterine scar defects, we established specific criteria for surgical laparoscopic repair. This procedure was exclusively recommended for individuals who exhibited large niches—specifically, those where the residual myometrium was less than 2.5 mm. Furthermore, candidates must have actively expressed a desire to conceive and demonstrated symptoms related to the niche. While our approach may not align perfectly with the latest recommendations from the CSDi Study Group published in 2023 [27], it is important to note that at the time of our study’s planning, our indications were grounded in the available research, which consisted of small treatment groups and limited follow-up analyses.

The consensus from the CSDi Study Group emphasizes that a diagnosis of CSDi does not necessarily mean that treatment is indicated, highlighting the need for a careful, individualized approach. We recognize that randomized controlled trials addressing these criteria are currently underway [28,29], and we look forward to incorporating their findings to refine our understanding of treatment indications in the future.

### Scar reconstruction

In our research, postoperative scar thickness increased to an average of 6.5 mm in TVU and 6.1 mm in HySoG. Similar results were observed in the studies conducted by Karampelas et al. [20] (6.7 mm) and Vervoort et al. [22] (5.3 mm) after 3–6 months. Donnez et al. [19] reported significantly higher values of 9.7 mm after 3 months.

In our study, the incidence of failure (RM < 2.5 mm) was 8.2% (n = 9). The findings of Karampelas et al. [20] showed that only 4.3% of cases with a scar <3 mm (1 out of 23), while Vervoort et al. [22] reported this situation in 11.9% of their patients. Donnez et al. [19] observed no cases of RM < 3 mm in the MRI assessment. Possible differences in the postoperative assessment methods might account for the varying results observed in the available studies. In our study, during follow-up, we found that in 33 cases, cervical mucus filled the canal and scar area, rendering HySoG inapplicable. The inconsistent results observed in the statistical analyses, which compared two-stage (TVU and HySoG) and single (TVU) examinations, underscore the need for further analysis of the efficacy of postoperative scar assessment.

The frequency of diverticulum occurrence after the repair procedure was 49% in our study, which had not been assessed in any previous studies. The depth of postoperative niche was presented by Vervoort et al. [22] and was found to be similar to what we obtained, with values of 2.7 mm (1.4–4.7) in TVU and 4.0 mm (2.2–6.3) in HySoG. Such a high frequency of diverticulum occurrence in our material and a relatively large depth after repair procedures in both studies can be explained by the natural process of uterine muscle scar healing. Scar tissue contraction pulls the uterine muscle from the cervical canal side, and often results in a defect in the mucous membrane layer along the entire scar, as evidenced by ultrasonographic examinations. Additionally, the reoccurrence of a small diverticulum in half of patients supports the opinion the ultrasound

### Surgical technique

In our study, the technique of two-layer suturing with horizontal mattress sutures in the first layer (three H-sutures with a second layer or two H-sutures with a second layer) proved to be optimal for repairing uterine scar defects. This technique resulted in the smallest depth of postoperative diverticulum and the greatest increase in the thickness of the reconstructed scar. This suggests that the horizontal mattress sutures used as the first layer better capture the base of the wound towards the cervical canal side and adapt its edges more effectively. The second layer contributes to the increase in scar thickness by bringing together the edges of the uterine wall. The favourable effect of this technique is likely due to the horizontal mattress sutures causing less ischemia to the opposing edges, and the second layer of sutures additionally reduces tension on the primary layer. The choice between two or three sutures depended on the width of the defect and the width of the excised scar, so the treatment outcomes did not differ between them.

Our study found that the reparative procedure was most successful during the follicular phase of the menstrual cycle, excluding the ovulatory period. Suture failures were less likely to occur and better RM outcomes were achieved during this phase. It can be suggested that the longer a time from the surgical repair to the following menstruation, i.e., until uterine hyperaemia and uterine contractions occur, the more favourable effect on healing is seen. In our study, patients were not referred for hormonal contraception immediately after the procedure, unlike in other studies [20–22]. However, the repair failure rate might be caused by other reasons as retroversion of the uterus, use of medication, smoking, collagen abnormalities and others.

### Pregnancy outcomes

Out of 127 women who underwent surgical treatment, only 62.2% were followed up in their attempts to become pregnant, as many abandoned their pregnancy plans immediately after the procedure. This limited the follow-up analysis and revealed how much age of the patients at diagnosis (1/3 were over 35 years old), long time from the last caesarean section to diagnosis (this was over 4 years), and the length of convalescence (6 months) diminish the desire to conceive. This study highlights the importance of considering factors beyond surgery in understanding fertility outcomes. Future research should consider these factors to improve surgical treatments’ effectiveness for fertility.

In our study of 79 patients trying to conceive, a significant number of women with CSD were able to successfully achieve their pregnancy goals. 62.0% of patients conceived and 53.2% gave birth to at least one child after undergoing surgical repair. Notably, 75% of the patients with CSD were primiparae, consistent with previous studies [22]. Scar repair procedures helped these patients overcome the challenge of a sizable scar defect in the uterus and successfully achieve their pregnancy goals. However, the type of surgical procedure did not influence the likelihood of conception or successful childbirth, as no statistically significant differences were observed.

### Main findings

1. The laparoscopic repair of scar niche substantially relieved symptoms associated with CSD and effectively enhanced measurable scar parameters.
2. The majority of patients who had a sustained desire to conceive were able to fulfill their aspirations after the surgery.
3. The choice of surgical technique has a bearing on the depth and frequency of postoperative defects as well as the thickness of the postoperative scar. The most favourable results were achieved with double-layer suturing and a horizontal mattress suture used in the first layer.
4. The optimal surgical outcomes are obtained when the procedure is performed during the follicular phase of the menstrual cycle before the periovulatory time.

To conclude, laparoscopic repair of Cesarean scar defect has been found to be effective in reducing clinical symptoms, particularly postmenstrual spotting, by up to 4 days and uterine bleeding length by up to 5 days. Additionally, it has been observed that the procedure has improved scar parameters with a failure rate of 8.2%. The intervention has resulted in nearly 60% success rates in achieving successful reproductive plans. However, the prolonged duration of the procedure has led to a notable proportion of patients abandoning their childbearing plans. Current research indicates that the most beneficial surgical technique for closing uterine wall incisions following complete resection of CSD involves a double-layer horizontal mattress suture. It is also recommended that the procedure should be performed during the preovulatory cycle phase. Careful selection of the surgical technique and timing of the procedure can significantly enhance the success rates of the intervention while minimizing the risks and complications associated with the surgery.

Finally, we would like to underscore the necessity of meticulous patient selection for laparoscopic repair of uterine scar defects in clinical practice. This approach is critical, as it addresses the significant concern regarding the potential for overtreatment. Current literature highlights the need for more substantial data on the obstetric implications of these niches, especially given the high prevalence of the scar disorder. Our study also suggests that, despite meticulous resuturing techniques, there remains a notable incidence of small niche formation, which may not be classified as a failure of the approach. By prioritizing careful patient selection and fostering further research, we can enhance clinical practices and patient outcomes in this area.

## Supporting information

S1 Fig Flowchart detailing the selection of patients for the cesarean section scar laparoscopic repair. (TIFF)

S1 Table The description of the laparoscopic niche resection and uterus repair. (DOCX)

S2 Table The impact of three types of uterine sutures (single layer cross-mattress sutures (X, single), double-layer cross-mattress sutures (X, double) or double-layer horizontal mattress sutures (H, double)) on the postoperative scar parameters (niche formation (post-NICHE) and residual myometrium (post-RM)) in transvaginal ultrasound (TVU) and hysterosalpingography (HySoG). Mean ±SD (n). med. (ICR1-ICR3). n (%).
(DOCX)

## References

[1] Jordans IPM, de Leeuw RA, Stegwee SI, Amso NN, Barri-Soldevila PN, van den Bosch T, et al. Sonographic examination of uterine niche in non-pregnant women: a modified Delphi procedure. Ultrasound Obstet Gynecol. 2019;53(1):107–15. doi: 10.1002/uog.19049 29536581 PMC6590297

[2] Bij de Vaate AJM, Brölmann HAM, van der Voet LF, van der Slikke JW, Veersema S, Huirne JAF. Ultrasound evaluation of the Cesarean scar: relation between a niche and postmenstrual spotting. Ultrasound Obstet Gynecol. 2011;37(1):93–9. doi: 10.1002/uog.8864 21031351

[3] van der Voet LF, Bij de Vaate AM, Veersema S, Brölmann HAM, Huirne JAF. Long-term complications of caesarean section. The niche in the scar: a prospective cohort study on niche prevalence and its relation to abnormal uterine bleeding. BJOG. 2014;121(2):236–44. doi: 10.1111/1471-0528.12542 24373597

[4] Abacjew-Chmyłko A, Wydra DG, Olszewska H. “Niche” czyli ubytek w miejscu blizny mięśniówki macicy po cięciu cesarskim - przyczyny, diagnostyka, objawy. Ginekol Pol. 2016;87(2):143–7. doi: 10.17772/gp/60072 27306292

[5] Verberkt C, Lemmers M, de Vries R, Stegwee SI, de Leeuw RA, Huirne JAF. Aetiology, risk factors and preventive strategies for niche development: A review. Best Pract Res Clin Obstet Gynaecol. 2023;90:102363. doi: 10.1016/j.bpobgyn.2023.102363 37385157

[6] Yao W, Chen Y, Yao H, Yao Q, Wang L, Wang M, Yue J. Uterine niche is associated with adverse in vitro fertilization and intracytoplasmic sperm injection outcomes: a retrospective cohort study. Fertil Steril. 2023 Mar;119(3):433–41. doi: 10.1016/j.fertnstert.2022.12.001 36493872

[7] Lawrenz B, Melado L, Garrido N, Coughlan C, Markova D, Fatemi H. Isthmocele and ovarian stimulation for IVF: considerations for a reproductive medicine specialist. Hum Reprod. 2020;35(1):89–99. doi: 10.1093/humrep/dez241 31885047

[8] Jurkovic D. Cesarean scar pregnancy and placenta accreta. Ultrasound Obstet Gynecol. 2014;43(4):361–2. doi: 10.1002/uog.13346 24692219

[9] Tulandi T, Cohen A. Emerging Manifestations of Cesarean Scar Defect in Reproductive-aged Women. J Minim Invasive Gynecol. 2016;23(6):893–902. doi: 10.1016/j.jmig.2016.06.020 27393285

[10] Jauniaux E, Chantraine F, Silver RM, Langhoff-Roos J, FIGO Placenta Accreta Diagnosis and Management Expert Consensus Panel. FIGO consensus guidelines on placenta accreta spectrum disorders: Epidemiology. Int J Gynaecol Obstet. 2018;140(3):265–73. doi: 10.1002/ijgo.12407 29405321

[11] de Vaate AJMB, Brölmann HAM, van der Slikke JW, Wouters MGAJ, Schats R, Huirne JAF. Therapeutic options of caesarean scar pregnancy: case series and literature review. J Clin Ultrasound. 2010;38(2):75–84. doi: 10.1002/jcu.20648 19902518

[12] Bujold E, Jastrow N, Simoneau J, Brunet S, Gauthier RJ. Prediction of complete uterine rupture by sonographic evaluation of the lower uterine segment. Am J Obstet Gynecol. 2009;201(3):320.e1–6. doi: 10.1016/j.ajog.2009.06.014 19733288

[13] Tanos V, Toney ZA. Uterine scar rupture - Prediction, prevention, diagnosis, and management. Best Pract Res Clin Obstet Gynaecol. 2019;59:115–31. doi: 10.1016/j.bpobgyn.2019.01.009 30837118

[14] Jordans IPM, Vissers J, de Leeuw RA, Hehenkamp WJK, Twisk JWR, de Groot CJM, et al. Change of the residual myometrial thickness during pregnancy in women who underwent laparoscopic niche resection compared with controls without niche surgery: a prospective comparative cohort study. Am J Obstet Gynecol. 2022;227(6):901.e1-901.e12. doi: 10.1016/j.ajog.2022.07.011 35841936

[15] Donnez O. Cesarean scar disorder: Management and repair. Best Pract Res Clin Obstet Gynaecol. 2023;90:102398. doi: 10.1016/j.bpobgyn.2023.102398 37598564

[16] Jacobson MT, Osias J, Velasco A, Charles R, Nezhat C. Laparoscopic repair of a uteroperitoneal fistula. JSLS. 2003;7(4):367–9. 14626405 PMC3021339

[17] Donnez O, Jadoul P, Squifflet J, Donnez J. Laparoscopic repair of wide and deep uterine scar dehiscence after cesarean section. Fertil Steril. 2008;89(4):974–80. doi: 10.1016/j.fertnstert.2007.04.024 17624346

[18] Marotta M-L, Donnez J, Squifflet J, Jadoul P, Darii N, Donnez O. Laparoscopic repair of post-cesarean section uterine scar defects diagnosed in nonpregnant women. J Minim Invasive Gynecol. 2013;20(3):386–91. doi: 10.1016/j.jmig.2012.12.006 23357466

[19] Donnez O, Donnez J, Orellana R, Dolmans M-M. Gynecological and obstetrical outcomes after laparoscopic repair of a cesarean scar defect in a series of 38 women. Fertil Steril. 2017;107(1):289-296.e2. doi: 10.1016/j.fertnstert.2016.09.033 27816234

[20] Karampelas S, Salem Wehbe G, de Landsheere L, Badr DA, Tebache L, Nisolle M. Laparoscopic Isthmocele Repair: Efficacy and Benefits before and after Subsequent Cesarean Section. J Clin Med. 2021;10(24):5785. doi: 10.3390/jcm10245785 34945080 PMC8708618

[21] Zhang N-N, Wang G-W, Zuo N, Yang Q. Novel laparoscopic surgery for the repair of cesarean scar defect without processing scar resection. BMC Pregnancy Childbirth. 2021;21(1):815. doi: 10.1186/s12884-021-04281-8 34879840 PMC8653604

[22] Vervoort A, Vissers J, Hehenkamp W, Brölmann H, Huirne J. The effect of laparoscopic resection of large niches in the uterine caesarean scar on symptoms, ultrasound findings and quality of life: a prospective cohort study. BJOG. 2018;125(3):317–25. doi: 10.1111/1471-0528.14822 28703935 PMC5811817

[23] Fabres C, Arriagada P, Fernández C, Mackenna A, Zegers F, Fernández E. Surgical treatment and follow-up of women with intermenstrual bleeding due to cesarean section scar defect. J Minim Invasive Gynecol. 2005;12(1):25–8. doi: 10.1016/j.jmig.2004.12.023 15904593

[24] Klemm P, Koehler C, Mangler M, Schneider U, Schneider A. Laparoscopic and vaginal repair of uterine scar dehiscence following cesarean section as detected by ultrasound. J Perinat Med. 2005;33(4):324–31. doi: 10.1515/JPM.2005.058 16207118

[25] Yalcinkaya TM, Akar ME, Kammire LD, Johnston-MacAnanny EB, Mertz HL. Robotic-assisted laparoscopic repair of symptomatic cesarean scar defect: a report of two cases. J Reprod Med. 2011;56(5–6):265–70. 21682124

[26] Huirne JAF, Vervoort AJMW, Leeuw RD, Brölmann HAM, Hehenkamp WJK. Technical aspects of the laparoscopic niche resection, a step-by-step tutorial. Eur J Obstet Gynecol Reprod Biol. 2017;219:106–12. doi: 10.1016/j.ejogrb.2017.09.019 29101836

[27] Klein Meuleman SJM, Murji A, van den Bosch T, Donnez O, Grimbizis G, Saridogan E, et al. Definition and Criteria for Diagnosing Cesarean Scar Disorder. JAMA Netw Open. 2023;6(3):e235321. doi: 10.1001/jamanetworkopen.2023.5321 36988956 PMC10061236

[28] Huirne JAF. LAPRESS study. February 20, 2017. Updated August 18, 2022. [Accessed February 23, 2023]. https://www.clinicaltrialregister.nl/nl/trial/28650

[29] Huirne JAF. Symptomatic niches for laparoscopic niche resection, a randomized clinical trial. June 11, 2021. [Accessed February 23, 2023]. https://onderzoekmetmensen.nl/en/trial/54858

