# Supplementary figures and images for: Laparoscopic repair of the caesarean section scar niche: A prospective cohort study
Source: PLoS One. 2025 Jul 2;20(7):e0318592. doi: 10.1371/journal.pone.0318592 (PMC12220985; doi:10.1371/journal.pone.0318592)

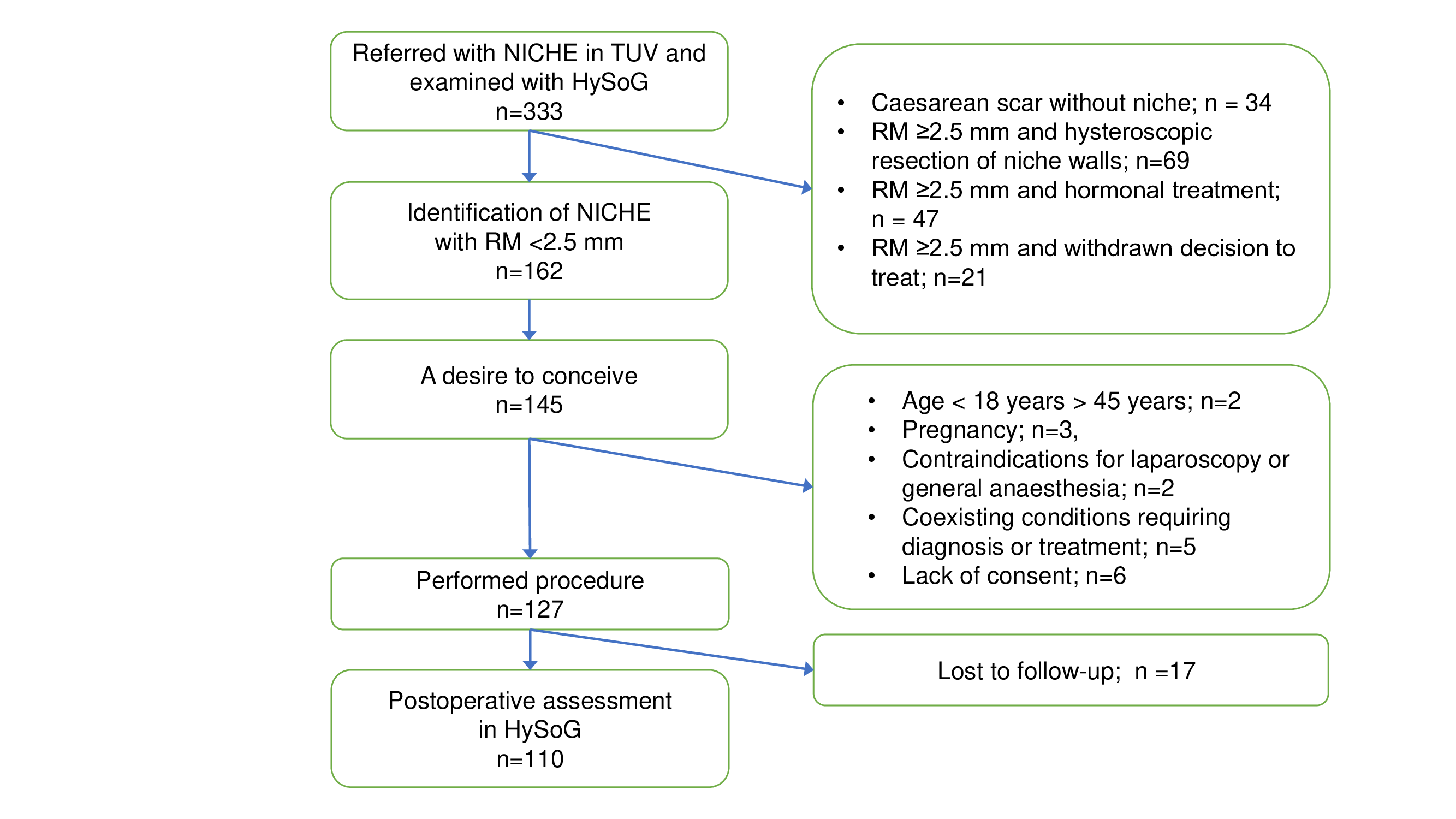

Supplement: S1 Fig — (TIFF) [file pone.0318592.s001.tiff]
