# Supplementary material for: Laparoscopic repair of the caesarean section scar niche: A prospective cohort study
Source: PLoS One. 2025 Jul 2;20(7):e0318592. doi: 10.1371/journal.pone.0318592 (PMC12220985; doi:10.1371/journal.pone.0318592)
Supplement: S1 Table — (DOCX) [file pone.0318592.s002.docx]

S1 Table. The description of the laparoscopic niche resection and uterus repair.

| Procedure | Description / Comment |
| --- | --- |
| 1. Identification of the niche | Insertion of a vaginal speculum and application of a tenaculum on the anterior lip of the cervix. Insertion of a uterine probe into the caesarean scar defect (in cases of uncertainty, identifying the defect using a hysteroscope). |
| 2. Insertion of a urinary catheter | - |
| 3. Laparoscopic adominal access | Introduction of trocars (one in the umbilicus, two laterally above the pubic bone, the third either laterally from the umbilicus or above the pubic bone), inspection of the pelvis and abdominal cavity (with identification of endometriosis foci), dissection of adhesions preventing access to the lower part of the uterus (peritoneal adhesions, adhesions of the omentum, and adhesions of the uterus with the anterior abdominal wall). Adhesiolysis of dense adhesions. |
| 4. Access to the uterine scar | Surgical incision of the peritoneal fold and dissection of a frequently adherent bladder from the lower uterine segment. This dissection aimed to create a clearance of about 1-1.5 cm below the scar from the previous caesarean section. The dissection of the bladder dependes on the degree of adhesions; for smaller adhesions, it was initiated in the midline, while for larger adhesions, it was started laterally creating paravesical spaces. The uterine vessels are optionally identified. |
| 5. Removal of the uterine defect | The repeated identification of the defect with a probe, incision of the uterus above the thinnest point of the defect, and excision of the remaining scar above the niche and excision of the proximal and distal walls of the scar pouch with laparoscopic cold scissors, including any retention cysts if present; the thickness of the excised margin of the scar defect depended on the extent of scar tissue - the aim was to achieve: (a) a normally appearing, slightly bleeding myometrium, (b) myometrium with full wall thickness on the proximal and distal sides of the uterus; electrocoagulation was only used locally in case of excessive bleeding; |
| 6. Reconstruction of the uterine wall | Use of two 1.0 dissolving sutures along the probe inserted into the cervical canal; use of two technique variations:   - 1. A single layer cross-mattress sutures (X sutures) covering the full thickness of the myometrium (including the endometrium);   2. A double layer suture: the first layer included ¾ of the deeper uterine wall thickness, along with the endometrial lining, while the second layer included the outer 1/4 - 1/3 of the thickness (the layers could overlap). Two or three deep sutures were applied using two techniques: cross-mattress sutures (X-sutures) and horizontal mattress sutures (H-sutures).   The second layer was performed using simple sutures. Knots were tied after the whole lines of stitches were completed. |
| 7. Final check | The integrity of the sutures was checked using a uterine probe. A bladder integrity test was conducted if there were doubts about the continuity of the bladder walls. |
