# Supplementary material for: Laparoscopic repair of the caesarean section scar niche: A prospective cohort study
Source: PLoS One. 2025 Jul 2;20(7):e0318592. doi: 10.1371/journal.pone.0318592 (PMC12220985; doi:10.1371/journal.pone.0318592)
Supplement: S2 Table — Mean ±SD (n). med. (ICR1-ICR3). n (%). (DOCX) [file pone.0318592.s003.docx]

**S2 Table. The impact of three types of uterine sutures (single layer cross-mattress sutures (X, single), double-layer cross-mattress sutures (X, double) or double-layer horizontal mattress sutures (H, double)) on the postoperative scar parameters (niche formation (post-NICHE) and residual myometrium (post-RM)) in transvaginal ultrasound (TVU) and hysterosalpingography (HySoG).**

| **Suturing**  **features**  **Scar feature** | **Technique** | | | | **Deep layer sutures** | | |
| --- | --- | --- | --- | --- | --- | --- | --- |
|  | **H, double** | **X, double** | **X, single** | ***P*-value** | **H** | **X** | ***P*-value** |
| post-NICHE >3mm (%) | 6  (21.4%) | 39  (58.2%) | 9  (60.0%) | **<0.001** | 6  (21.4%) | 48  (59.3%) | **<0.001** |
| post-NICHE >2mm (%) | 14  (50.0%) | 53  (79.1%) | 11  (73.3%) | **0.02** | 14  (50.0%) | 64  (79.0%) | **0.003** |
| post-NICHE depth, mm | 2.4 ±1.6 (28) | 4.4 ± 2.6 (67) | 4.2 ±3.0  (15) | **0.003** | 2.4 ±1.6  (28) | 4.3 ±2.7  (82) | **<0.001** |
| post-NICHE depth TVU, mm | 2.0 ±1.5 (28) | 3.5 ±2.5  (66) | 3.6 ±2.3  (15) | **0.01** | 2.0 ±1.6  (28) | 3.5 ±2.5  (81) | **0.003** |
| post-NICHE depth HySoG, mm | 2.6 ±1.8 (16) | 4.8 ±2.6  (50) | 4.6 ±3.2  (11) | **0.02** | 2.62 ±1.85  (16) | 4.8 ±2.8  (61) | **0.003** |
| post-RM thickness total, mm | 6.3 ±2.1 (28) | 6.1 ±2.7  (68) | 5.9 ±0.2  (14) | 0.9 | 6.3 ±2.1  (28) | 6.1 ±2.7  (82) | 0.7 |
| post-RM thickness TVU, mm | 6.5 ±2.4 (28) | 6.5 ±2.8  (67) | 6.4 ±2.8  (14) | 0.9 | 6.5 ±2.4  (28) | 6.5 ±2.7  (81) | 1.0 |
| post-RM thickness HySoG  mm | 6.6 ±1.8 (16) | 6.0 ±2.5  (51) | 6.0 ±0.2 (10) | 0.6 | 6.6 ±1.8  (16) | 6.0 ±2.5  (61) | 0.3 |
| post-RM <2.5 mm (%) | 1  (3.6%) | 7  (10.2%) | 2  (14.3%) | 0.3 | 1  (3.6%) | 9  (11.0%) | 0.3 |
| post-RM <4 mm (%) | 3  (10.7%) | 16 (23.5%) | 4  (28.6%) | 0.2 | 3  (10.7%) | 20 (24.4%) | 0.1 |
| ∆ post-RM – pre-RM thickness TVU, mm | 4.5 ±2.2 (28) | 4.1 ±2.8 (68) | 3.6 ±3.4 (14) | 0.7 | 4.5 ±2.2 (28) | 4.0 ±2.9 (82) | 0.5 |
| ∆ post-RM – pre-RM thickness HySoG, mm | 5.3 ±2.3  (16) | 4.6 ±2.6 (52) | 4.7 ±2.7 (10) | 0.6 | 5.3 ±2.3  (16) | 4.7 ±2.6 (62) | 0.5 |

Mean ±SD (n)

med. (ICR1-ICR3)

n (%)
